# Acute effects of resistance exercise intensity and repetition at a predetermined volume on inhibitory control: a randomized controlled trial

**DOI:** 10.3389/fspor.2025.1551624

**Published:** 2025-03-13

**Authors:** Ying-Chu Chen, Yan-Ho Lo, Chen-Sin Hung, Yi-Ting Cheng, Ruei-Hong Li, Feng-Tzu Chen, Yu-Kai Chang

**Affiliations:** ^1^ Department of Physical Education and Sport Sciences, National Taiwan Normal University, Taipei, Taiwan; ^2^ Department of Kinesiology, National Tsing Hua University, Hsinchu, Taiwan; ^3^ Social Emotional Education and Development Center, National Taiwan Normal University, Taipei, Taiwan

**Keywords:** acute exercise, physical activity, resistance exercise, cognitive function, executive function

## Abstract

**Background:**

This study explores the effects of acute resistance exercise (RE) on inhibitory control (IC), emphasizing exercise volume.

**Methods:**

In total, 78 young adults were randomly assigned to one of three groups: the moderate-intensity group [MI; 60% one repetition maximum (1RM), 3 × 10 reps], the low-intensity group (LI; 30% 1RM, 3 × 20 reps), or the control group (CON; 35 min reading). The exercise groups were volume-matched. Baseline equivalence was assessed via one-way analysis of variance (ANOVA) for demographic variables and chi-square for gender. One-way analysis of covariance examined post-test interreference inverse efficiency score scores, controlling for pre-test values. Two-way ANOVA assessed Group (MI, LI, CON) × Time effects on heart rate (resting; pre-test, during intervention, and post-test), rate of perceived exertion (pre-test, during intervention, and post-test), and lactate (pre-test, mid-test, and post-test). Mean differences and 95% confidence intervals supplemented *p*-values.

**Results:**

IC was assessed using the Stroop Task, revealing better performance in both the MI (*p* = 0.026) and LI (*p* = 0.040) groups compared to CON, though no significant difference was found between the two exercise groups. Blood lactate levels significantly increased post-exercise in both the MI (*p* = 0.012) and LI (*p* < 0.001) groups, but again, there was no significant difference between them.

**Conclusions:**

These findings highlight that acute RE, regardless of intensity, enhances IC and raises blood lactate when exercise volume is controlled. Practitioners might tailor RE protocols by adjusting the intensity to match individuals’ capabilities without compromising the cognitive and physiological benefits.

**Clinical Trial Registration:**

identifier (NCT05311202).

## 1 Introduction

Executive function is a higher-order cognitive process that regulates lower-level cognitive operations to support goal-directed behavior (1, 2). Superior executive function is associated with improved mental health (3–5), physiological wellbeing (6, 7), quality of life (8, 9), career performance (10, 11), and brain health (12). A key executive function, inhibitory control (IC), facilitates the suppression of irrelevant stimuli, enabling focus on target goals (13, 14). It plays a crucial role in emotional regulation (15, 16), impulse control (17), and goal-directed behavior (18), making it fundamental to cognitive function.

Acute exercise—a single bout of exercise—has been shown to enhance executive function (19–22), primarily through mechanisms such as increased brain-derived neurotrophic factor (BDNF) release, which promotes synaptic plasticity and neuronal survival (23), and reduced central sensitization, optimizing cognitive processes (24). While most studies focus on aerobic exercise (25–28), limited research examines the impact of resistance exercise on executive function (29). A meta-analysis of 12 studies reported a moderate to large effect (effect size = 0.73) of acute resistance exercise on inhibitory control (30), a finding corroborated by a systematic review (29). The 2018 Physical Activity Guidelines for Americans also highlight the cognitive benefits of acute exercise, including resistance training (31).

Beyond confirming the benefits of acute and chronic resistance exercise on inhibitory control (32, 33), research has explored how resistance exercise parameters such as intensity, sets, and repetitions influence cognitive outcomes. Brush et al. (34) found that high-intensity resistance exercise reduced Stroop task reaction time, while Tsukamoto et al. (35) reported greater improvements in inhibitory control following high-intensity resistance exercise compared to low-intensity, despite identical total repetitions. However, emerging evidence suggests that total exercise volume—rather than intensity alone—may be the primary driver of cognitive benefits (36). Their study examined volume-matched protocols across different intensities and indicated that lower-volume resistance exercise might benefit higher-order cognitive functions, while higher-volume exercise was more effective for lower-order cognitive domains (37). However, variations in rest intervals and the absence of control conditions limit the generalizability of these findings. Tomoo et al. (38) further explored intensity-matched resistance exercise effects but faced similar limitations due to small sample sizes and the lack of control groups (CON).

Despite these insights, few studies have examined moderate-intensity resistance exercise [50%–69% one repetition maximum (1RM), 8–12 repetitions] within a controlled framework, despite its established benefits for health and muscular fitness (39). Previous research is constrained by inconsistent exercise prescriptions, small sample sizes, and the absence of non-exercise control conditions, making it difficult to isolate the specific role of moderate-intensity resistance training in improving inhibitory control. Addressing this gap, our study systematically investigated the acute effects of moderate-intensity, volume-matched resistance exercise on inhibitory control, aligning with American College of Sports Medicine (ACSM) guidelines. By employing a larger sample, incorporating a control group, and standardizing total exercise volume, this study aimed to refine our understanding of how resistance exercise intensity and volume interact to influence executive function. We hypothesized that moderate-intensity resistance exercise would yield greater improvements in inhibitory control than a non-exercise control group, while volume-matching across intensities would produce comparable cognitive benefits. This study advances the field by providing a clearer framework for optimizing resistance exercise to enhance cognitive performance.

## 2 Material and methods

### 2.1 Participants and sampling

Recruitment and the first measurements for this study started in April 2022 and were conducted using the university newsletter and flyer. The last measurements were conducted in December 2022. The required sample size was determined through an *a priori* power analysis using G*Power 3.1 [power = 0.80, alpha = 0.05, effect size *f* = 0.2 (30)], which indicated a minimum of 66 participants. To account for potential attrition and ensure sufficient statistical power, the recruitment target was increased to 84 participants. The study eventually included 78 young adults aged from 20 to 26 years old. The eligibility criteria for this research comprised the following: (1) had no history of brain-related disorders; were not taking medications affecting brain function; did not have epilepsy; was devoid of any prior cardiovascular, cerebrovascular, or respiratory ailments; and not under any cardiovascular medication; (2) individuals possessing normal or corrected eyesight; without color blindness or color weakness; (3) subjects self-reporting as having no prior regular exercise routine over the previous 6 months, assessed through the International Physical Activity Questionnaire Screening; and (4) individuals lacking musculoskeletal disorders that might hinder engagement in physical activities. Before baseline assessment, all participants had to fill out the Physical Activity Readiness Questionnaire (PAR-Q).

Grounded in a rigorous experimental framework, the study protocol was duly registered on ClinicalTrials.gov (prospective registration identifier: NCT05311202). In addition, we adhered to the Consolidated Standards of Reporting Trials (CONSORT) statements (40) and the Template for Intervention Description and Replication (TIDieR) checklist (41) to ensure transparent and comprehensive reporting of our methods and findings. Upon careful review of our study procedures, we acknowledged a minor deviation from the originally registered protocol, specifically in the number of intervention groups. This adjustment was made to better explore the potential role of resistance exercise volume while ensuring the study's scientific integrity. The modification was carefully considered and did not compromise the validity of the findings. The study was granted approval by the Research Ethics Committee of National Taiwan Normal University (202201HM012, approval date: 15 February 2022).

All measurements were conducted following approval. This research adhered to the standards outlined in the International Conference on Harmonization guidelines for Good Clinical Practice and the Declaration of Helsinki. Before participating, all individuals provided written informed consent after receiving a full explanation of the study and were free to withdraw from the experiment at any time without providing a reason.

### 2.2 Exercise intervention

**Figure 1:**
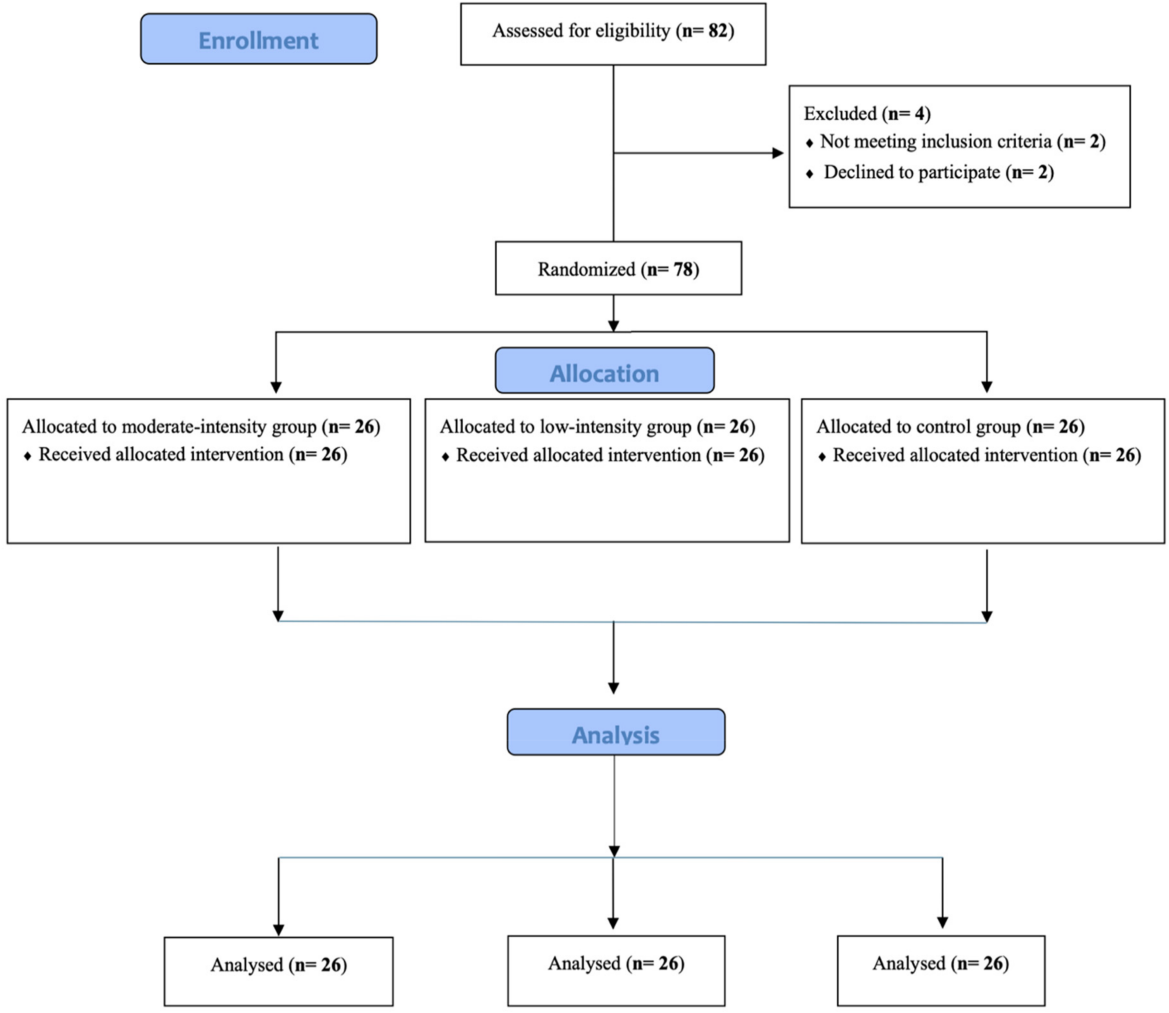
CONSORT 2010 flow diagram.

This was a single-center, open-label, randomized controlled trial with three parallel arms conducted at National Taiwan Normal University. Between-subjects pre-test–post-test comparisons were utilized and participants were randomly assigned to the following three parallel groups with an allocation ratio of 1:1:1 by a blind outcome adjudicator to avoid the potential risk of bias: (1) the low-intensity exercise group (LI) who performed resistance exercises at an intensity of 30% 1RM for three sets of 20 repetitions each (34); (2) the moderate-intensity exercise group (MI) who engaged in resistance exercises at an intensity of 60% 1RM for three sets of 10 repetitions each (39); (3) the CON who read. In this parallel design randomized controlled trial, we used the CONSORT 2010 flow diagram, as illustrated in Figure 1, and participants were allocated to one of three groups (LI, MI, or CON) through stratified randomization, ensuring gender balance across groups for comparability. However, due to the study design, neither participants nor assessors were blinded to group assignments. The training volume for the exercise interventions was calculated based on the resistance exercise recommendations of the ACSM (39).

The resistance exercise intervention was administered by an National Strength and Conditioning Association-Certified Personal Trainer (NSCA-CPT), a credentialed professional with expertise in designing and implementing safe and effective strength training programs. The trainer possessed a strong background in exercise science, including knowledge of biomechanics, physiology, and individualized programming. To ensure consistency and adherence to the study protocol, the trainer underwent specific training on the intervention protocol, including standardized exercise prescription, participant monitoring, and adherence strategies, thereby maintaining the reliability and effectiveness of the resistance exercise intervention. During the resistance exercises, the trainer delivered the one-on-one and face-to-face instruction in the weight-training room on the basement floor of the National Taiwan Normal University. In addition, this study employed four compound resistance exercises—the Smith machine bench press, Smith machine bent-over row, Smith machine squat, and Smith machine deadlift—as interventions. There was a 2-min rest interval between sets for each exercise, maintaining a consistent rhythm during movements (1-s concentric contraction and 2-s eccentric contraction). The exercise intervention durations were 40 min for the LI group, 35 min for the MI group, and 35 min for the CON group.

### 2.3 Experimental procedure

The participants attended for a total of 2 days, with at least 7-day intervals between each. On the first day of the lab visit, participants completed questionnaires covering demographic information and the PAR-Q and were briefed on the experiment, including the assessment for 1RM.

Prior to each intervention session, the participants were instructed to abstain from vigorous exercise for 24 h and to avoid alcohol, caffeine, medications, and ergogenic aids. These measures were undertaken to minimize potential confounding influences on both exercise capacity and cognitive performance, given that caffeine and other stimulants can modulate reaction speed and attention (42). The testing environment was meticulously controlled, maintaining minimal noise levels and a temperature range of 24°C–26°C. The intervention days involved either the exercise interventions or a control intervention. Before the intervention, the participants had their lactate levels measured and performed the Stroop Color World Task (SCWT). The participants were briefed on the post-test and ensured they reached an 85% familiarity with the tasks before undergoing a total of three SCWT blocks (43).

**Figure 2:**
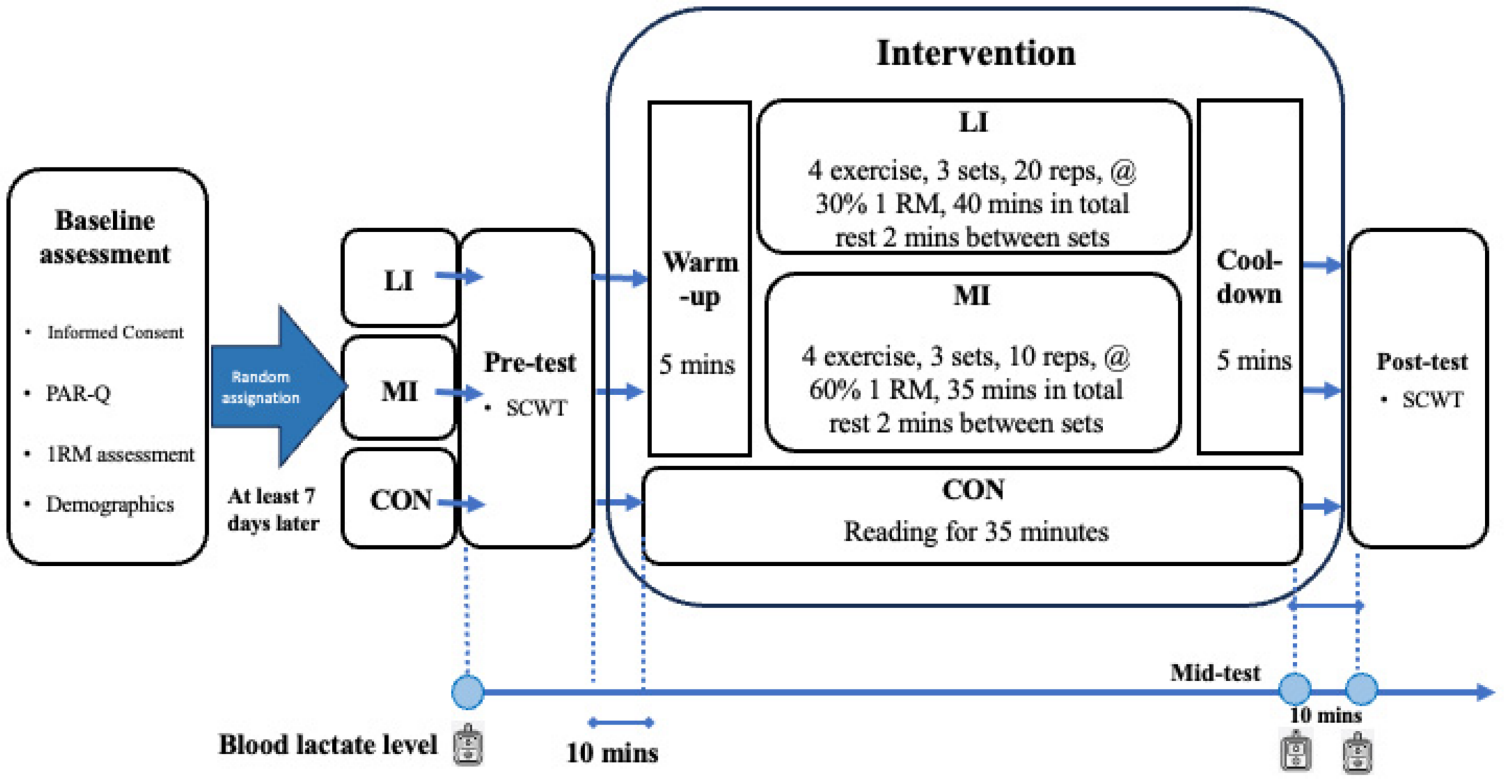
Experimental procedure. CON, control group; LI, low-intensity exercise group; MI, moderate-intensity exercise group; PAR-Q, Physical Activity Readiness Questionnaire; RM, repetition maximum; SCWT, Stroop Color World Task; reps, repetitions; mins, minutes.

Before the pre-test SCWT, blood lactate levels were measured. After completing the test, the participants moved to the gymnasium, engaged in a 5-min warm-up, followed by approximately 35 or 40 min of resistance training, and concluded with a 5-min cool-down. After the intervention, blood lactate levels were measured immediately as mid-test and 10 min later as post-test. Subsequently, a post-test SCWT was administered. The experimental procedure is presented in Figure 2.

### 2.4 Manipulation check parameters

The manipulation check parameters included heart rate, blood lactate level, and rate of perceived exertion (RPE). Heart rate was measured using a Polar H10 chest strap (Polar Electro Oy, Kempele, Finland). Fingertip blood samples were collected to determine blood metabolite responses. Blood lactate level (mmol/L) was measured using a lactate analyzer (EDGE: Blood Lactate Monitoring System, Hsinchu Science Park, Hsinchu, Taiwan, China). In the case of RPE, the Borg psychophysiological rating (1–10 scale) (44, 45) was applied to assess individuals’ subjective psychophysiological feelings regarding their RPE.

### 2.5 1RM strength assessment

The resistance exercise assessment protocol comprised several key steps. Participants initiated the protocol with a light-intensity warm-up, followed by a 1-min rest interval. For the initial set of exercises, the workload was incrementally increased by 5%–10% for upper body exercises and 10%–20% for lower body exercises. Participants were then instructed to perform 10 repetitions, followed by a 2-min rest interval. Subsequently, the load was progressively increased until only a single repetition could be performed. This process determined the 1RM, representing the maximal load an individual could lift for one repetition, across four exercises, including the Smith machine bench press, Smith machine bent-over row, Smith machine squat, and Smith machine deadlift. A similar assessment procedure has been utilized in several studies (46, 47).

### 2.6 Stroop Color World Task

In the assessment of inhibitory control, the computerized Chinese iteration of the SCWT, as delineated in preceding studies (43, 48), was employed. The implementation of this task was facilitated through the utilization of NeuroScan STIM2 software, developed by Neurosoft Labs Inc. The stimuli encompassed three categories, namely neutral, congruent, and incongruent stimuli. The congruent and incongruent stimuli featured three Chinese words [“紅 (RED),” “綠 (GREEN),” and “藍 (BLUE)”] displayed in a 2 × 2 cm font size, presented centrally for a duration of 500 ms against a black background. The fixed inter-stimulus interval was set at 2,000 ms.

Each stimulus was rendered in one of the three specified colors, namely red (RGB: 254, 0, 0), green (RGB: 255, 200, 25), and blue (RGB: 0, 0, 254). Consequently, the stimuli were semantically congruent with the color of the word (e.g., the word “RED” printed in red for congruent trials) or incongruent with it (e.g., the word “RED” printed in blue for incongruent trials). Participants were explicitly directed to engage in a choice reaction time task, wherein they were required to promptly and accurately press one of the three color-labeled keys on the keyboard corresponding to the printed colors of the stimuli. Only accurate responses falling within the designated response time window (i.e., 200–1,200 ms following the onset of the initial stimulus) were considered for analysis. Each stimulus type was presented randomly and with equal probability across three blocks, each comprising 72 trials. The inverse efficiency score (IES) was initially computed by dividing the mean response time of correct trials by accuracy [IES = reaction time/(1 − proportion of errors) = reaction time/proportion of correct response]. Subsequently, the interreference score of IES (IIES) was derived by subtracting the IES of the congruent condition from that of the incongruent condition. As the IIES decreases, inhibitory control performance decreases (49, 50).

### 2.7 Statistical analysis

Demographic data were presented as mean ± standard deviation. Outliers, defined herein as observations with a standardized *Z*-score exceeding ±3.29, were adjusted by assigning a raw score for the respective variable that deviated by one unit from the next most extreme score within the distribution. Normality was assessed using the standardized *Z*-score, with values falling outside the range of ±1.96 indicating a potential deviation from normal distribution. In instances of violation, the raw scores underwent either square root or logarithmic transformation. Should normality assumptions persistently remain unmet post-transformation, the Kruskal–Wallis test was employed for non-parametric analysis (51).

To assess baseline equivalence among groups, one-way analyses of variance (ANOVAs) and the chi-square test were employed for the demographic data [i.e., age, height, weight, body mass index (BMI), memory span (forward and backward), and 1RM and workload for the four exercises] and gender, respectively. One-way analysis of covariance (ANCOVA) was conducted to examine the exercise effect on the post-test of IIES, with the pre-test controlled as a covariate. Two-way ANOVA was utilized to analyze the Group (three groups: MI, LI, and CON) and Time effects of heart rate (HR: resting HR, pre-test HR, intervention HR, and post-test HR), RPE (pre-test RPE, intervention RPE, and post-test RPE), and lactate (pre-test lactate, mid-test lactate, and post-test lactate). To complement the *p*-values, mean differences and the 95% confidence interval (CI) were reported for each outcome. If the sphericity assumption was violated, the Greenhouse–Geisser correction was applied. SPSS 29.0 statistical software was applied for data analysis, and the statistical significance level was set at *⍺* = 0.05.

## 3 Results

### 3.1 Data screen, transformation, and analyses of demographic data

Age, height, weight, BMI, the digit span backward test, and squat 1RM each conformed to a normal distribution. Consequently, log transformations were applied to the digit span forward test, deadlift 1RM, bench press 1RM, rowing 1RM, and total workload for the four exercises. Despite these transformations, the rowing 1RM and the workloads for the deadlift and bench press remained non-normally distributed, necessitating the use of the Kruskal–Wallis test.

One-way ANOVA revealed that the main effect of Group was not significant for age, height, weight, BMI, digit span backward test, deadlift 1RM, bench press 1RM, squat 1RM, and squat and rowing workloads (*p*s > 0.05). The Kruskal–Wallis test indicated that rowing 1RM and deadlift and bench press workloads were not different between the groups (*p*s > 0.05). The chi-square test indicated that gender was not different among the groups, with means and standard deviations shown in Table 1.

**Table 1:** Demographics and health data of the participants (mean ± standard deviation).

Parameter	MI (*n* = 26)	LI (*n* = 26)	CON (*n* = 26)
Gender (M/F)	16/10	14/12	15/11
Age (year)	22.62 ± 1.92	22.62 ± 1.86	22.77 ± 1.82
Height (cm)	163.71 ± 8.88	168.25 ± 9.16	166.14 ± 9.54
Weight (kg)	58.64 ± 9.77	62.77 ± 8.62	60.54 ± 10.67
BMI (kg/m^2^)	21.77 ± 2.29	22.15 ± 2.33	21.80 ± 2.27
Digit span test			
Forward^a^	15.00 ± 1.10	14.96 ± 1.46	15.35 ± 0.94
Backward	10.38 ± 2.65	9.92 ± 2.92	10.69 ± 2.85
1RM (lb)			
Deadlift^a^	106.08 ± 49.00	106.50 ± 46.13	101.19 ± 33.73
Bench press^a^	66.67 ± 35.20	71.60 ± 38.26	69.73 ± 34.20
Squat	113.06 ± 37.89	110.45 ± 44.52	122.69 ± 43.41
Bent-over row^b^	74.22 ± 32.17	73.83 ± 27.04	71.44 ± 20.95
Workload			
Deadlift^b^	1,985.10 ± 1,045.57	1,883.08 ± 805.41	—
Bench press^b^	1,286.63 ± 842.74	1,339.96 ± 652.57	—
Squat^a^	2,108.44 ± 793.76	1,985.62 ± 797.34	—
Bent-over row^a^	1,388.08 ± 680.38	1,381.04 ± 652.33	—

M, male; F, female; CON, control group; LI, low-intensity exercise group; MI, moderate-intensity exercise group; BMI, body mass index; RM, repetition maximum.

^a^ Indicates that the data represent the raw score, but the *p*-value was obtained from an ANOVA performed after the log transformation of the score.

^b^ Indicates that the data represent the raw score, but the *p*-value was obtained using the Kruskal–Wallis test.

### 3.2 Inhibitory control

**Figure 3:**
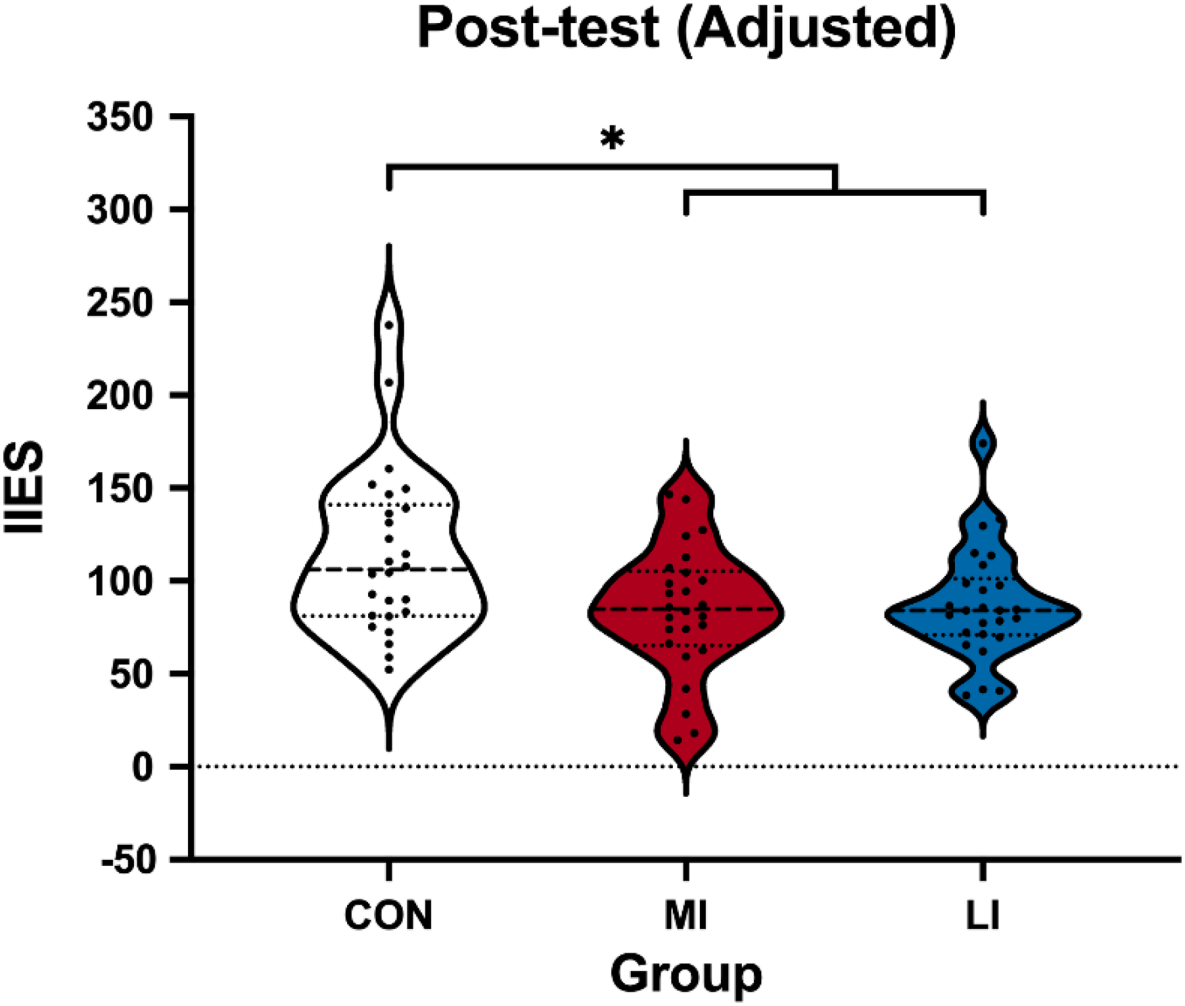
Violin plot of adjusted post-test IIESs among the groups. IIES, interreference inverse efficiency score; CON, control group; MI, moderate-intensity exercise; LI, low-intensity exercise group. The recorded post-test IIES was a raw score and was adjusted using the pre-test IIES. *p < 0.05.

The one-way ANCOVA revealed a significant main effect of Group (*F*_2,74_ = 4.55, *p* = 0.014, $\eta_{p}^{2}$  = 0.11), and the pairwise comparison revealed that the IIES of both the MI (87.34 ± 7.23 ms; *p* = 0.026) and LI groups (87.34 ± 7.23 ms; *p* = 0.040) was smaller than the CON group (114.07 ± 7.29 ms), with mean differences of 26.72 ms (95% CI 1.50–51.95) for MI vs. CON and 29.95 ms (95% CI 4.58–55.33) for LI vs. CON. There was no difference between the MI and LI groups (mean difference = 3.23 ms; 95% CI −21.83 to 28.29; *p* = 1.000] (Figure 3).

### 3.3 Exercise manipulation check

#### 3.3.1 Heart rate

The normality assumption for the pre-HR values was initially violated but met after log transformation. To maintain consistency across units, all HR values underwent log transformation for statistical analysis. Two-way ANOVA indicated a significant main effect of Group (*F*_2,75_ = 15.52, *p* < 0.001, $\eta_{p}^{2}$ = 0.29), Time (*F*_2.37,177.47_ = 160.73, *p* < 0.001, $\eta_{p}^{2}$ = 0.61), and Group × Time interaction (*F*_4.73,177.47_ = 57.34, *p* < 0.001, $\eta_{p}^{2}$ = 0.61).

The simple main effect of Group revealed no difference in HR among the groups at rest (MI: 76.69 ± 2.22 bpm; LI: 75.69 ± 2.22 bpm; CON: 74.62 ± 2.22 bpm; *p*s > 0.05) and pre-test (MI: 83.89 ± 2.05 bpm; LI: 83.12 ± 2.05 bpm; CON: 84.23 ± 2.05 bpm; *p*s > 0.05). During the intervention, both the MI (117.52 ± 2.76 bpm; mean difference = 42.90 bpm, 95% CI 33.35–52.46; *p* < 0.001) and LI groups (117.11 ± 2.76 bpm; mean difference = 42.50 bpm, 95% CI 32.95–52.05; *p* < 0.001) had significantly higher HRs compared to the CON group (74.62 ± 2.76 bpm), with no difference between the MI and LI groups (mean difference = −0.41 bpm, 95% CI −9.96 to 9.14; *p* = 1.000). At post-test, both the MI (88.92 ± 2.05 bpm; mean difference = 11.06 bpm, 95% CI 3.97–18.14; *p* < 0.001) and LI groups (87.04 ± 2.05 bpm; mean difference = 9.17 bpm, 95% CI 2.09–16.26; *p* = 0.007) had significantly higher HRs compared to the CON group (77.87 ± 2.05 bpm), with no difference between the MI and LI groups (mean difference = −1.89 bpm, 95% CI 8.97–5.20; *p* = 1.000).

The simple main effect of Time revealed that HR was highest during the intervention and post-test, followed by pre-test and resting (*p*s < 0.001; mean difference between intervention and resting = 42.90 bpm, 95% CI 33.35–52.46; *p* < 0.001; mean difference between post-test and resting = 11.06 bpm, 95% CI 3.97–18.14; *p* < 0.001), with no difference between the intervention period and post-test (mean difference = −1.89 bpm, 95% CI −8.97 to 5.20; *p* = 0.44). In the LI group, HR was highest during the intervention and post-test, followed by pre-test and resting (*p*s < 0.001; mean difference between intervention and resting = 42.50 bpm, 95% CI 32.95–52.05; *p* < 0.001; mean difference between post-test and resting = 9.17 bpm, 95% CI 2.09–16.26; *p* = 0.007), with no difference between the intervention period and post-test (mean difference = −1.89 bpm, 95% CI −8.97 to 5.20; *p* = 1.000).

In the CON group, HR at resting was significantly lower than at pre-test (mean difference = −9.17 bpm, 95% CI −16.26 to −2.09; *p* = 0.007), but there were no differences compared to the intervention (mean difference = −42.90 bpm, 95% CI −52.46 to −33.35; *p* = 1.000) and post-test (mean difference = −11.06 bpm, 95% CI −18.14 to −3.97; *p* < 0.001). Pre-test HR was significantly lower than during the intervention (mean difference = −42.90 bpm, 95% CI −52.46 to −33.35; *p* < 0.001) and post-test (mean difference = −11.06 bpm, 95% CI −18.14 to −3.97; *p* < 0.001). There was no difference in HR at mid- and post-test (mean difference = −1.89 bpm, 95% CI −8.97 to 5.20; *p* = 1.000).

#### 3.3.2 Blood lactate level

The normality assumption for blood lactate levels was initially violated across tests but met after log transformation. Two-way ANOVA revealed a significant main effect of Group (*F*_2,75_ = 850.53, *p* = 0.002, $\eta_{p}^{2}$ = 0.16), Time (*F*_2,150_ = 72.72, *p* < 0.001, $\eta_{p}^{2}$ = 0.49), and Group × Time interaction (*F*_4,150_ = 14.82, *p* < 0.001, $\eta_{p}^{2}$ = 0.28).

The simple main effect of Group revealed that there was no pre-test difference in blood lactate levels among the groups (MI: 2.65 ± 0.37; LI: 2.79 ± 0.37; CON: 3.74 ± 0.37; *p*s > 0.05). At mid-test, both the MI (8.25 ± 0.74; mean difference = 3.84, 95% CI 1.28–6.39; *p* = .001) and LI groups (9.25 ± 0.74; mean difference = 4.84, 95% CI 2.29–7.40; *p* < 0.001) had significantly higher lactate levels compared to the CON group (4.41 ± 0.74), with no difference between the MI and LI groups (mean difference = −1.01, 95% CI −3.57 to 1.55; *p* = 1.000). At post-test, both the MI (5.48 ± 0.62; mean difference = 1.97, 95% CI −0.17 to 4.10; *p* = 0.081) and LI groups (7.61 ± 0.62; mean difference = 4.10, 95% CI 1.96–6.23; *p* < 0.001) had significantly higher lactate levels compared to the CON group (3.52 ± 0.62), with no difference between the MI and LI groups (mean difference = −2.13, 95% CI −4.26 to 0.002; *p* = 0.050).

**Figure 4:**
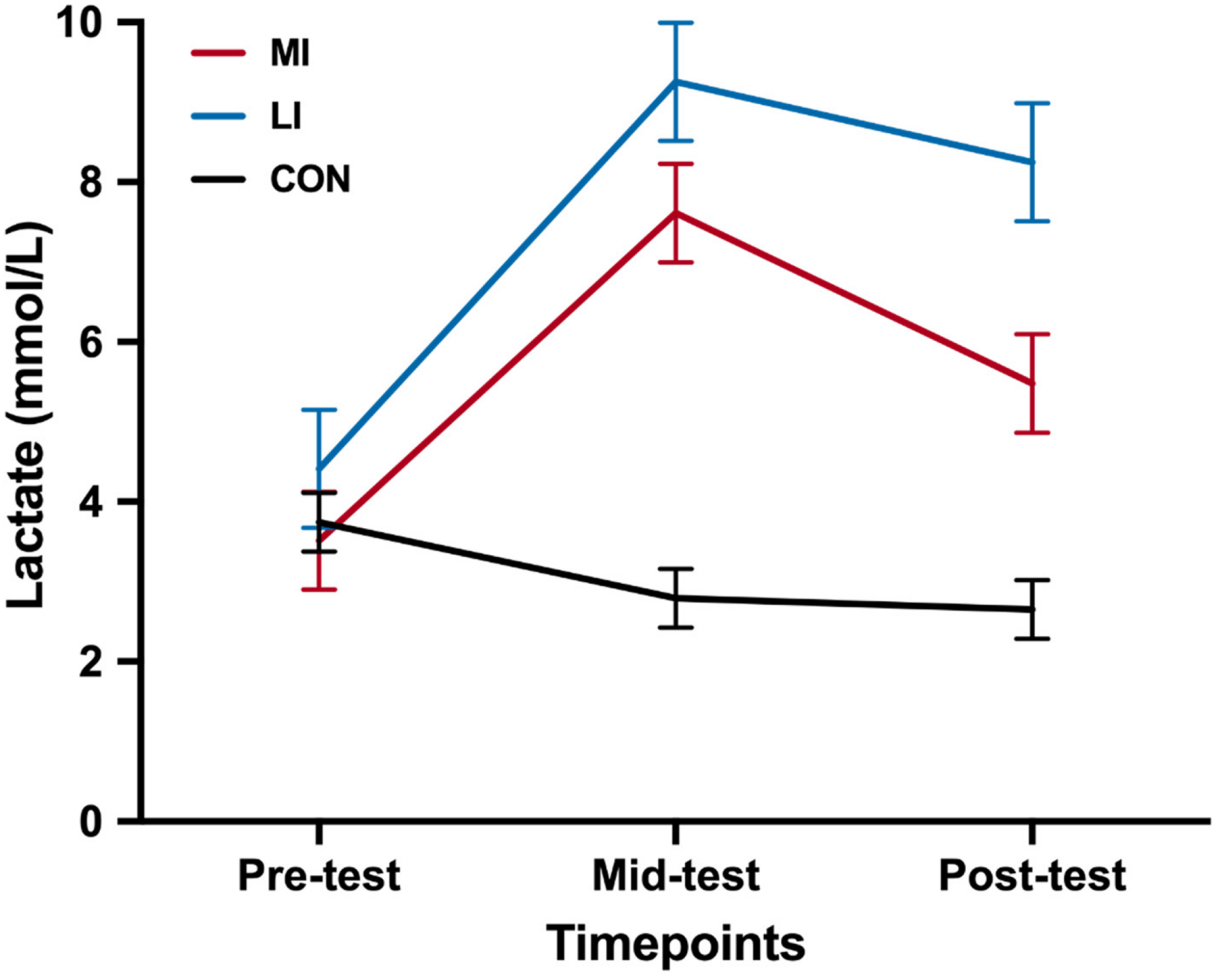
Lactate level at different timepoints among the groups. CON, control group; MI, moderate-intensity exercise; LI, low-intensity exercise group. *p < 0.05.

The simple main effect of Time revealed that the mid-test blood lactate levels were significantly higher, followed by post-test and pre-test in the MI group (*p*s < 0.001). In the LI group, both mid- and post-test blood lactate levels were significantly higher than at pre-test (*p*s < 0.001), but there was no difference between post- and mid-test (mean difference = −2.13, 95% CI −4.26 to 0.002; *p* = 0.050). In the CON group, there was no difference in blood lactate levels across the tests (*p*s > 0.05) (Figure 4).

## 4 Discussion

This study is the first to investigate the acute effects of resistance exercise on inhibitory control while examining the joint role of intensity and repetition at an ACSM-prescribed exercise volume. We hypothesized that participants performing moderate-intensity resistance exercise would show greater gains in inhibitory control than a non-exercise control group and that, while total training volume is held constant, varying intensity and repetition would yield similar cognitive benefits. Our results supported the general notion that acute resistance exercise enhances inhibitory control compared to a non-exercise control group. Notably, this benefit was observed at equivalent exercise volumes regardless of whether moderate-intensity or low-intensity resistance exercise protocols were employed, indicating that altering load and repetition within the same total volume does not attenuate the cognitive gains. Moreover, both the moderate- and low-intensity groups exhibited comparable increases in lactate levels at mid- and post-test, suggesting a similar acute metabolic response potentially underpinning these improvements in inhibitory control.

Taking into consideration both our findings and earlier investigations employing crossover or parallel group designs, it appears that a wide range of resistance exercise intensities—spanning approximately 40%–90% of 1RM—can elicit improvements in inhibitory control (37). Notably, much of the existing evidence is grounded in Stroop Task paradigms, which necessitate controlled processing by a central executor (34, 37, 52). Within this broad spectrum, a more refined threshold zone of around 70% 10RM (∼53% 1RM) to 80% 1RM has been posited as potentially optimal for enhancing higher-order cognitive functions (35). In alignment with these observations, our results corroborate previous studies indicating that moderate-intensity resistance training, anchored in ACSM guidelines—typically 50%–69% 1RM (39)—significantly augments inhibitory control (36, 38, 46, 47, 53). Collectively, these convergent findings underscore the efficacy of moderate-intensity protocols for fostering executive function improvements, particularly in the domain of inhibitory control.

Different from moderate intensity, studies have observed limited effects for low-intensity resistance exercise. Building on this investigation, Chang and Etnier (52) found an inverted U-shaped dose–response relationship between acute resistance exercise intensity (low, moderate, and high) and inhibitory control performance, with moderate intensity (70% 10RM, ≈53% 1RM) showing more benefits than low intensity. Correspondingly, Engeroff et al. (37) indicated that a conceivable threshold window for achieving maximal benefits can be delineated between approximately 70% 10RM (approximately 53% 1RM) and 80% 1RM. Similarly, Brush et al. (34) found no immediate effects for low intensity (40% 10RM). This notwithstanding, our study focused not only on resistance exercise intensity but also on exercise volume. That is, our study aimed to further elucidate the acute effects of low- and moderate-intensity resistance exercise on inhibitory control when exercise volume is carefully controlled, in which exercise volume simultaneously involves intensity and repetition. Our results demonstrated that once the pre-determined volume is met, an acute exercise effect is observed regardless of exercise intensity. In line with Tomoo et al. (38), who showed that both low-intensity (35% 1RM) and high-intensity (70% 1RM) volume-matched bilateral leg extension resistance exercises enhance inhibitory control, our results further suggest that intensities as low as 30% 1RM can also produce comparable benefits to those of moderate intensities (60% 1RM) when training volume is held constant. Importantly, our findings extend Tomoo et al.'s work by demonstrating that even lower loads—30% of 1RM—can elicit improvements in executive function. This underscores the feasibility of prescribing relatively light resistance exercise to achieve cognitive benefits and thereby broadens the application of acute resistance exercise for populations who may be unable or unwilling to train at higher intensities.

Regarding the potential mechanism, from a psychosocial perspective, factors such as the placebo and contextual effects may contribute to the observed improvements in inhibitory control. The placebo effect, which has been widely studied in exercise and motor performance research, suggests that cognitive benefits may be influenced by participants’ expectations regarding exercise outcomes (54). Given that resistance exercise is often associated with enhanced cognitive function, participants in the exercise conditions may have an internal or external focus of attention on anticipated performance improvements, thereby amplifying the effects. Similarly, the contextual effect—the impact of the exercise environment, researcher–participant interactions, and the structured nature of an experimental setting—could have potentially influenced outcomes (55).

Beyond these psychosocial factors, physiological mechanisms also play a critical role in mediating the cognitive benefits of resistance exercise. While expectations and environmental factors may contribute to enhanced inhibitory control, resistance exercises typically involve energy expenditure and limited blood flow during periods of muscle tension, triggering physiological responses that further support cognitive improvements. Resistance exercises typically involve energy expenditure and limited blood flow during periods of muscle tension (56). The hypoxic conditions within the exercising muscle necessitate a reliance on anaerobic energy metabolism, which becomes a crucial component during resistance exercise. That is, lactate level may help interpret the relationship between resistance exercise total volume and inhibitory control performance since lactate is one of the exercise-induced myokines and exerkines that may play a pivotal role in muscle–brain cross-talk (57–59). The lactic acid system functions as an anaerobic pathway in the human body's energy metabolism. During a resistance exercise session, lactate substitutes glucose as the primary energy source for the brain (60), while the supply of oxygen in the blood is insufficient to meet the demand due to increased muscle tension from skeletal muscle contractions. These contractions compress micro vessels, reducing oxygen delivery to the muscles and decreasing the ability to remove lactate from the blood (61). Moreover, there is an increased recruitment of type II muscle fibers during resistance exercise, which results in greater oxygen utilization for the breakdown of glucose to produce additional energy (62). The body's oxygen supply falls short of demand, prompting the utilization of the lactate system, which leads to the accumulation of lactate in the blood (63), and further facilitates the potential cross-talk signaling between the muscle and the brain (64).

Our study found similar increases in blood lactate levels in both the MI (60% 1RM) and LI groups (30% 1RM) compared to the CON group following interventions with the same training volume. Although blood lactate concentration is positively correlated with exercise intensity, blood lactate levels may also be influenced by various parameters, including exercise volume, the extent and size of the recruited muscle mass, and the load and speed of execution (65). Tomoo et al. (38) demonstrated that high-volume low-intensity (35% 1RM) of six sets and 20 repetitions induced higher blood lactate levels than high-intensity (70% 1RM) of six sets and 10 repetitions in volume-matched acute bilateral leg extension resistance exercise. Similarly, Dora et al. (53) demonstrated that very slow tempo low-intensity (30% 1RM) resistance exercise (3 s eccentric and concentric contraction and 1 s isometric contraction) results in sustained higher blood lactate levels 10 min post-exercise, compared to a normal tempo low-intensity (30% 1RM) intervention (1-s eccentric, concentric, and isometric contraction). Although these two previous studies produced blood lactate levels that differ from those observed in our study, both studies demonstrated a similar pattern in inhibitory control performance at the 10-min post-test mark. Consequently, the degree of post-exercise peripheral biomarkers, such as blood lactate levels, but not the level induced by different exercise intensities directly influence the inhibitory control performance observed following acute resistance exercise. Since our study controlled for total exercise volume and observed similar metabolic responses across exercise groups, it is likely that both physiological and psychological mechanisms collectively contribute to the observed cognitive gains.

Different exercise modalities produce distinct acute physiological responses (e.g., muscle damage, tension, fatigue, and metabolic stress), which may influence inhibitory control performance (22, 66). A key distinction between our study and that of Tomoo et al. (38) and Dora et al. (53) lies in the type of resistance exercises used. Multi-joint exercises engage multiple muscle groups, requiring greater neuromuscular coordination, balance, and motor planning, making them cognitively more demanding than single-joint exercises (22). Exercise-cognition research suggests that higher task complexity can enhance cognitive benefits by increasing attentional demands and executive control (67, 68). Our findings provide new evidence that multi-joint exercises can be as effective as, or even more stimulating than, single-joint exercises for improving inhibitory control. Practically, exercise selection should consider individual and contextual factors (e.g., training goals, equipment availability, and movement specificity), while recognizing the role of task complexity in cognitive outcomes. Future studies should further compare the effects of single-joint and multi-joint resistance exercises on inhibitory control while maintaining equal training volume across conditions.

## 5 Strengths and limitations

This study has several key strengths that contribute to the growing body of research on the acute cognitive effects of resistance exercise. First, it is the first investigation to systematically examine the joint influence of intensity and repetition while maintaining an ACSM-prescribed total exercise volume. By controlling for total training volume, our findings provide novel insights into the role of load and repetition in cognitive benefits, demonstrating that inhibitory control improvements occur regardless of whether resistance exercise is performed at a moderate or low intensity. This challenges previous assumptions that cognitive benefits are exclusive to moderate-intensity resistance exercise, expanding the applicability of resistance training for broader populations, including those with a limited capacity for higher-intensity training. Collectively, our study offers important implications for both research and clinical applications. By demonstrating that light-load resistance exercise can yield cognitive benefits equivalent to higher-intensity protocols when volume is controlled, we provide a feasible exercise prescription for populations who may not tolerate heavy resistance training. This expands the accessibility of resistance exercise as a cognitive intervention strategy, particularly for older adults, clinical populations, and those with limited strength training experience.

However, the study has limitations that warrant consideration. First, participants’ prior exercise experience was not controlled for, which could introduce variability in terms of fitness levels, movement familiarity, and lifting capacity. Second, the study concluded at the 10-min mark, preventing the assessment of longer-term effects and leaving the persistence of observed benefits beyond the immediate post-intervention period uncertain. Future research should extend follow-up intervals to evaluate the durability of these effects more comprehensively. Moreover, the study utilized only the midpoint of the ACSM-recommended training volumes, limiting the generalizability of the findings to other training regimens. To enhance the applicability of future research, it would be valuable to explore a broader array of ACSM-recommended training volumes and intensities to strengthen the real-world applicability of our findings. Incorporating biochemical measures—such as BDNF and insulin-like growth factor 1 (IGF-1)—would offer deeper insight into the physiological mechanisms underpinning these acute cognitive benefits.

Future studies should also extend their scope to populations with specific needs for enhanced executive functioning, including individuals with cognitive impairments or neurological conditions such as migraine. In addition, to further refine the methodological rigor and better isolate the true effects of acute resistance exercise, incorporating a placebo or sham exercise group in randomized controlled trials is recommended (69). By refining these protocols and examining their impact across diverse groups, research can better elucidate how resistance exercise might be optimally tailored to improve cognitive outcomes in those most likely to benefit.

The current study has important implications for both clinicians and researchers. Clinicians, particularly those working in exercise prescription and cognitive health, may consider incorporating resistance exercise as a non-pharmacological strategy to enhance inhibitory control, which is crucial for daily decision-making and self-regulation. The observed cognitive benefits, regardless of intensity when total exercise volume is controlled, suggest that individuals with varying physical capabilities—such as older adults or clinical populations—could achieve similar cognitive improvements by adjusting the load and repetitions to match their capacity. For researchers, these results highlight the need for further investigation into the underlying mechanisms linking acute metabolic responses to cognitive performance. Future studies should explore how different resistance exercise parameters influence cognitive function over time and whether similar effects extend to other cognitive domains beyond inhibitory control.

## 6 Conclusion

The current study suggests that moderate-intensity acute resistance exercise, as recommended by the ACSM, yields immediate benefits for inhibitory control. Furthermore, low-intensity acute resistance exercise has similar effects on inhibitory control performance when the pre-determined volume is met. In addition, it was observed that the physiological responses elicited by low and moderate-intensity acute resistance exercise, under the same training volume, were similar, as indicated by comparable increases in blood lactate concentration.

## Data Availability

The raw data supporting the conclusions of this article will be made available by the authors, without undue reservation.
